# “I had a knot in my heart”: unusual complication after implantation of a Swan-Ganz catheter

**DOI:** 10.1186/s12871-025-03317-2

**Published:** 2025-09-01

**Authors:** Zulfugar. T. Taghiyev, Andreas Böning

**Affiliations:** Department of Cardiovascular Surgery, Justus-Liebig University Hospital, Rudolf-Buchheim Str. 7, Giessen, 35392 Germany

**Keywords:** Knot, Swan-Ganz catheter, Pulmonary artery catheter, Complication

## Abstract

**Background:**

The implantation of a Swan-Ganz catheter for invasive hemodynamic monitoring is an established measure after cardiac surgery. A rare but serious complication is the formation of a knot in the heart, which can be diagnostically challenging. We report on a patient who developed left heart failure postoperatively after quadruple bypass surgery combined with aortic valve replacement and in whom a knot formed inadvertently during monitoring using a Swan-Ganz catheter.

**Case presentation:**

An 82-year-old female underwent combined quadruple coronary artery bypass grafting and aortic valve replacement for severe coronary and valvular heart disease. Postoperatively, she experienced acute left ventricular dysfunction, necessitating mechanical circulatory support with an Impella device. A Swan-Ganz catheter was placed through the internal jugular vein for accurate hemodynamic monitoring. After placement, unexpected catheter immobility raised suspicion of an intracardiac knot. Initial transthoracic echocardiography did not clearly visualize the lesion; however, subsequent chest radiography and jugular vein ultrasound confirmed catheter-associated intracardiac knot formation. Considering the heightened risk for cardiac injury and thromboembolic events, interdisciplinary consensus recommended bedside surgical extraction. The catheter and associated knot were successfully removed via transcutaneous vascular incision without complications. Inspection revealed knot formation proximal to the catheter thermistor, while the balloon remained intact and functional.

**Conclusion:**

Intracardiac knot formation associated with Swan-Ganz catheter placement is rare but presents significant risks. Timely interdisciplinary assessment, multimodal imaging, and surgical extraction under controlled conditions effectively mitigate potential complications, enabling safe catheter removal and favorable patient outcomes.

## Introduction

The Swan-Ganz catheter, developed in the 1970 s by Jeremy Swan and William Ganz, revolutionized hemodynamic monitoring of critically ill patients. The ability to measure cardiac output, intracardiac pressures, and systemic and pulmonary vascular resistance directly at the bedside represented a significant improvement over previous procedures that required patient treatment in catheter laboratories [1, 2]. Despite its high clinical importance, the Swan-Ganz catheter carries risks such as arrhythmias, infections, or rare complications such as intracardiac knot formation, which can lead to the need for significant therapeutic interventions [3, 4].

Notably, the ongoing application of the Swan-Ganz catheter in contemporary complex cardiac surgery exemplifies a clinical paradox, wherein its indispensable diagnostic and therapeutic value must be weighed against its inherent potential for severe complications.

## Case report

An 82-year-old female patient underwent quadruple coronary artery bypass grafting combined with aortic valve replacement due to severe coronary artery and valvular heart disease. There was an acute deterioration of the left ventricular pumping function postoperatively that necessitated the implantation of an Impella 5.5 heart pump. In order to ensure reliable hemodynamic monitoring, a Swan-Ganz catheter was subsequently implanted via the internal jugular vein separately during postoperative ICU care.

**Fig. 1:**
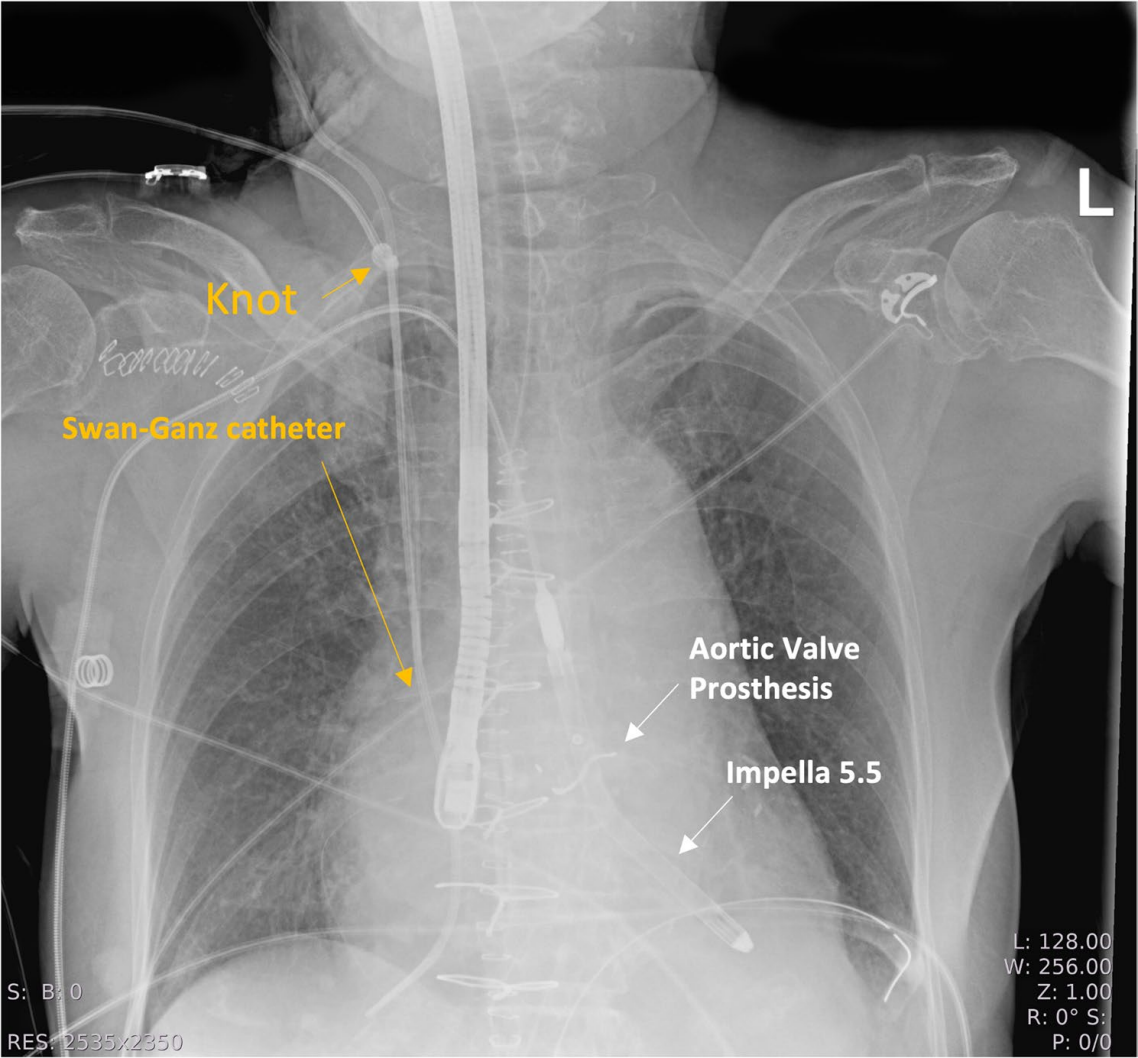
Chest X-ray

**Fig. 2:**
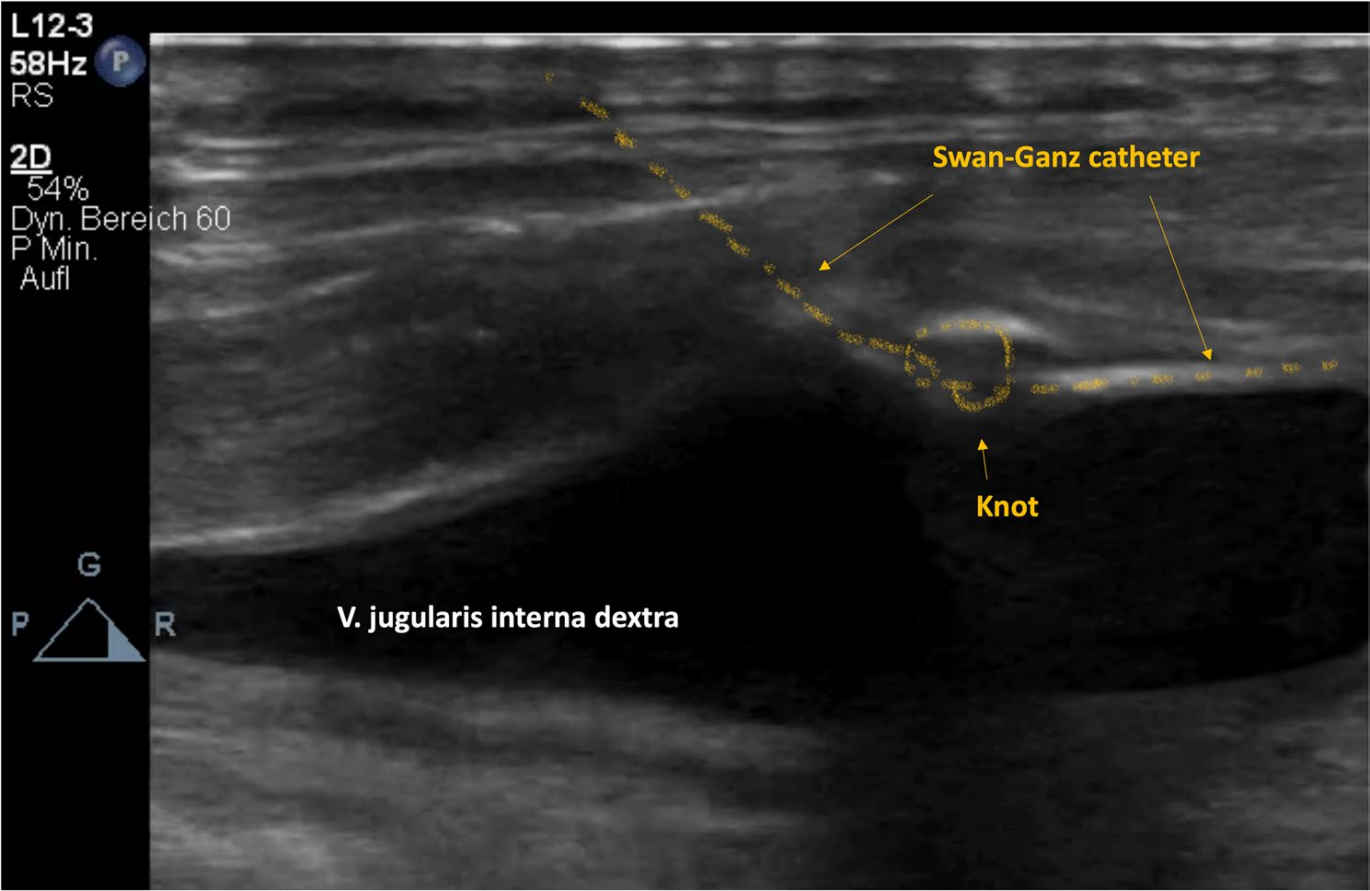
Ultrasound examination of the jugular vein

After initial placement, the Swan-Ganz catheter allowed for right ventricular pressure measurement; however, further advancement into the pulmonary artery to achieve wedge position was unsuccessful, necessitating several repositioning maneuvers. Following these attempts, a persistent blockade in catheter movement was encountered, especially during attempted withdrawal. Given the suspicion of intracardiac knot formation, transesophageal echocardiography was first performed, though it did not provide sufficient visualization of the catheter or knot. Subsequently, chest radiography was performed, which definitively confirmed the presence of a catheter-associated knot (Fig. 1). It is likely that repeated manipulations in the early phase resulted in malpositioning of the catheter tip into the inferior vena cava (IVC), as later demonstrated on imaging. A subsequent ultrasound examination of the jugular vein allowed clear visualization of the knot (Fig. 2).

The percutaneous surgical removal of the knot was performed via direct vascular access under controlled conditions. A parallel incision was made adjacent to the introducer sheath, and under gentle traction, the vessel was locally extended using a stab incision directly over the protruding knot. This maneuver enabled the simultaneous extraction of both the catheter and the knot, after which the vessel was closed with direct suturing. This technique was specifically chosen to minimize the risk of thromboembolism or cardiac injury. The procedure was successful without complications, and the patient subsequently showed stable cardiac function so that he could be discharged after careful monitoring.

**Fig. 3:**
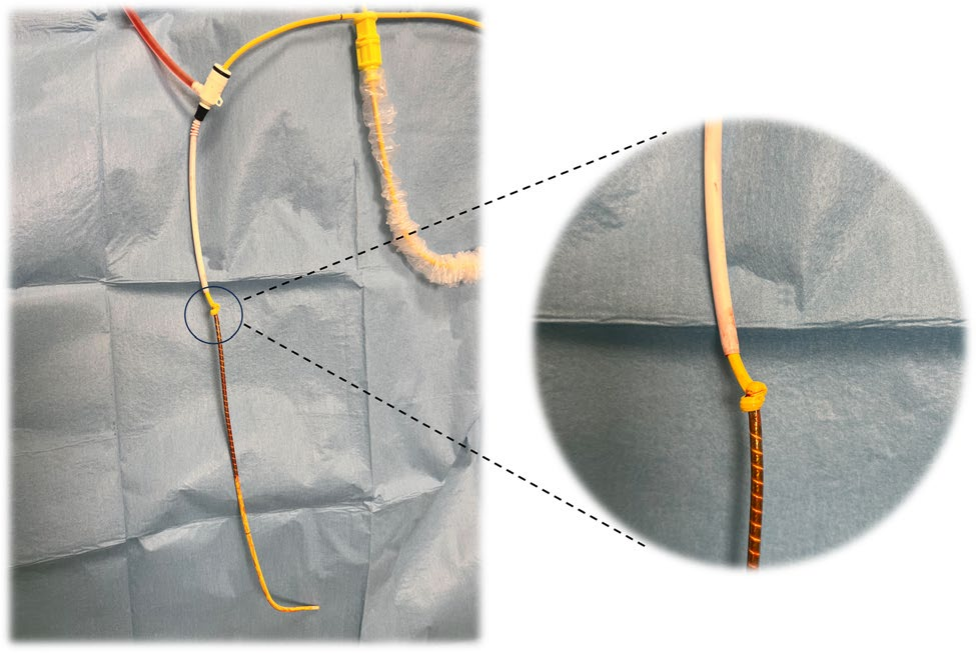
Detailed examination of the extracted Swan-Ganz catheter

A detailed examination of the extracted Swan-Ganz catheter showed knot formation just above the thermistor (Fig. 3), with the balloon remaining intact and fully functional at the catheter tip.

## Discussion

Knot formation in Swan-Ganz catheters often occurs as a result of excessive manipulation during insertion, such as repeated forward and backward movements of the catheter [3]. The risk is further increased by frequent adjustments and repositioning. The incidence of knotting ranges from 0.2–2.5%[5]. This complication, while rare, represents a significant challenge in hemodynamic monitoring, with multiple contributing factors related to technique, catheter design, and patient-specific factors.

In our case, the knot formation occurred immediately after placement and led to a complete impairment of the catheter movement. This resulted in the formation of a knot directly at the thermistor junction, a phenomenon that can be explained through several key mechanisms:

First, regarding insertion techniques, blind introduction and excessive manipulation during placement can lead to loops forming within the catheter [3, 6]. Our case clearly demonstrates this mechanism, as repeated adjustments during placement directly contributed to the rapid knot formation at the thermistor junction.

Second, the catheter’s inherent characteristics played a crucial role. The structural weakness at the thermistor junction in our patient likely predisposed to localized stress and knot formation. This observation aligns with well-documented vulnerabilities of Swan-Ganz catheters, where their long length and thin walls increase flexibility and susceptibility to knotting, particularly in dilated right heart chambers [3, 7].

Finally, the material properties themselves contributed to this complication. The differences in material properties at the thermistor junction may lead to loop formation and, in rare cases, solid knot formation during manipulation. This was strikingly evident in our case where the knot formed precisely at this junction despite maintained balloon function, while cracks developed proximally.

Remarkably, both the distal lumen and the lumen for inflation of the balloon at the catheter tip remained patent, enabling pressure measurements and contributing to initial diagnostic uncertainty. However, after removal and careful inspection, it was evident that fluid infused through the distal lumen exited through cracks proximal to the site of the knot, as well as at the tip. Therefore, our suspicion of kinking or knot formation was based exclusively on the observed blockade to catheter movement, rather than on pressure measurement abnormalities. This finding underscores the complex interplay between mechanical stress and material integrity in catheter-related complications. Additional environmental factors, such as heat-induced bending of the catheter and incomplete balloon inflation during advancement [6]while not directly observed in our case, further emphasize the importance of meticulous technique in preventing such complications.

Importantly, the presence of an additional central venous catheter in close proximity to the pulmonary artery sheath—clearly visible in the chest X-ray (Fig. 1) —may have contributed to directional changes at the Swan-Ganz catheter tip, potentially predisposing to looping or knotting, although direct interaction between the catheters would most likely occur within the superior vena cava due to the length of the introducer sheath.

The diagnosis of an intracardiac knot caused by a Swan-Ganz catheter is typically made in cases where there is difficulty in recording hemodynamics, in injecting fluids, or in moving the catheter [3]. In our patient’s case, echocardiography did not provide sufficient clarity, which often occurs with intracardiac knot formation. Therefore, supplementary diagnostics using chest X-ray and vascular ultrasound proved to be crucial.

Therapeutic measures recommended in the literature include non-surgical methods such as balloon dilation within the knot [8] or sling techniques with loop snare catheters [3, 9]. However, due to the patient’s stable condition, and in order to avoid possible complications such as cardiac injury or thromboembolic events, the decision was made to perform transcutaneous surgical removal with vascular incision and subsequent suturing.

## Conclusion

Early detection of intracardiac knot formation through imaging (chest X-ray, vascular ultrasound) is critical for timely intervention. While non-surgical retrieval techniques exist, our case demonstrates that surgical removal via vascular access may represent the safest approach in select scenarios, particularly when catheter integrity is compromised. This complication highlights three fundamental principles of management: minimizing catheter manipulation remains paramount for prevention, interdisciplinary collaboration proves essential for optimal approach selection, and vigilant monitoring is necessary to identify these rare but serious complications. Ultimately, the decision between interventional and surgical management must be individualized, carefully weighing procedural risks against patient-specific factors.

## Data Availability

No datasets were generated or analysed during the current study.

## References

[1] Keymel S, Steiner S. *Swan-Ganz Pulmonary Artery Catheterization-Safe But Not Harmless. *(2016).

[2] Neya K. Swan-Ganz catheter (pulmonary artery catheter). Kyobu Geka. 2009;62:677–81. 20715691

[3] Levin R, Vaca Valverde I, Porcile R. Resolución percutánea de Un Catéter de Swan-Ganz Anudado. Rev Argent Cardiol. 2019;87(4):319.

[4] Alconero Camarero AR, Gutiérrez Sandoval S, García Gómez V. *Complicaciones del catéter swan-ganz en pacientes diagnosticados de procedimientos quirúrgicos cardiovasculares. Enfermeria en Cardiologia*. 2008;44/2:29–32.

[5] Shang X, Yan Y, Wang B. Successful retrieval of a knotted Swan-Ganz catheter using interventional approach in an adult: a case report. Anaesthesiol Intensive Ther. 2016;48(5):367–70. 27785780 10.5603/AIT.a2016.0048

[6] Carrillo-Esper R, Visoso-Palacios P, Suárez-Mendoza C A. Anudamiento de Catéter de Swan-Ganz En La Rama derecha de La arteria pulmonar. Cir Cir. 2003;71(3):229–34. 14617412

[7] Dach J, Galbut D. The knotted swan-ganz catheter: new solution to a vexing problem. Am J Roentgenol. 1981;137(6):1274–5. 6976107 10.2214/ajr.137.6.1274

[8] Matta A, Boudou N, Ohayon P, Carrié D. Angioplasty inflated balloon to unknot an entrapped Swan-Ganz catheter. Eur Heart J - Case Rep. 2019;3(4):1–2. 32123794 10.1093/ehjcr/ytz190 PMC7042137

[9] Canitrot R, Lhermusier T, Servo C. *Complication of a Swan-Ganz catheter: an intravascular knot. *2023. 10.1093/ehjcr/ytad543 PMC10655053 38025121

